# Diphtheria outbreak in Nigeria: what we know now

**DOI:** 10.1016/j.infpip.2024.100345

**Published:** 2024-02-03

**Authors:** Daniel Danladi Gaiya, Paul Chijioke Ozioko, Moses Edache Entonu, Chioma U. Umeasiegbu

**Affiliations:** a Biology Unit, Air Force Institute of Technology, Nigerian Air Force Base, Kawo, Kaduna State, Nigeria; b School of Medical Biology, South Ural State University, Chelyabinsk, Russia

*Sir,*

The re-emergence of diphtheria in Nigeria calls for urgent action from the Nigeria Ministry of Health and other relevant health organizations to contain its spread. Diphtheria is a bacterial disease caused by *Corynebacterium diphtheriae*, which infects the respiratory tract, the nose and the skin [1]. Nasopharyngeal diphtheria is characterized by sore throat, swollen glands in the neck, weakness, obstruction of the airways, myocarditis, polyneuropathy and kidney failure. However, these symptoms depend on the part of the body that is affected. Symptoms develop within 2–5 days of onset in symptomatic patients [1,2]. *Corynebacterium diphtheriae* primarily infect the respiratory tract (throat, pharynx and nose) by attaching to the lining of the respiratory tract and releasing an extracellular protein called diphtheria toxin (DT) [3,4]. DT inhibits protein synthesis and kills susceptible healthy cells. The dead cells build up over time to form a thick, grey layer that can make it difficult to breathe and eat. The diphtheria toxin can also enter the bloodstream [4]. This can cause serious damage to the heart, kidneys and nerves and eventually lead to death [3].

In the wake of multiple outbreaks of diphtheria in some states of Nigeria (with the first confirmed case in the Federal Capital Territory (FCT), Abuja) there is a need for the Federal Government of Nigeria and the Nigeria Centre for Disease Control and Prevention (NCDC) to activate surveillance, hygiene and vaccination programmes to contain the spread of the disease [4]. Diphtheria, a vaccine-preventable disease, continues to be a serious problem in low-income countries due to low vaccination coverage. This is caused by non-adherence, lack of adequate vaccines, religious and cultural beliefs, vaccine fears, lack of adequate health facilities and poor environmental conditions [1,3]. It is the leading cause of child mortality in low-income and some developing countries [1].

On 1 December 2022, the Nigeria Centre for Disease Control and Prevention (NCDC) was notified of suspected diphtheria outbreaks in Kano and Lagos states. In response to the notification, rapid response teams (RRTs) were deployed to both states for outbreak confirmation and response support. Outbreaks of diphtheria have been confirmed in both states, and the NCDC is supporting the response activities in both states [5]. The cumulative report of epidemiological findings between week 19 of 2022 - week 03 of 2023, reported a total of 253 suspected cases: these were in Kano (169), Yobe (78), Lagos (5) and Osun (1) states [5]. Of the cases reported, eight were confirmed by laboratory tests (laboratory confirmed), 103 were found to have symptoms consistent with the clinical description of the diseases (clinically compatible), 18 were discarded, 40 are awaiting classification, while 84 were unknown [5]. The majority of cases occurred in children between 2 and 14 years of age. Among the cases, a total of 22 deaths were recorded [5,6].

In July 2023, a total of 1,506 suspected cases of diphtheria were reported from 59 Local Government areas (LGAs) in 11 states of the country [6]. Kano (1,055), Yobe (232), Kaduna (85), Katsina (58), Bauchi (47) and Federal Capital Territory (FCT) (18) accounted for 99.3% of all suspected cases. Most of the suspected cases reported were confirmed through laboratory testing and clinical evaluation of signs and symptoms that correlate with the clinical description of diphtheria disease. According to the Diphtheria Situation Report from the Nigeria Centre for Diseases Control and Prevention, a total of 4,160 suspected cases were reported from 27 states in 139 LGAs in Nigeria from May 2022 to July 2023. Of the 27 states where diphtheria was suspected, 87 cases were laboratory confirmed in 10 states, Kano 47(54.02%), Yobe 11(12.64%), Katsina 7(8.05%), Kaduna 5(7.75%), Bauchi 4(4.60%), FCT 4(4.60%) and Lagos 4(4.60%) account for 94.25% of suspected cases, while Niger 2(2.30%), Osun 1(1.15%) and Gombe 2(2.30%) account for the remaining 5.75% (Figure 1) [7].

**Figure 1:**
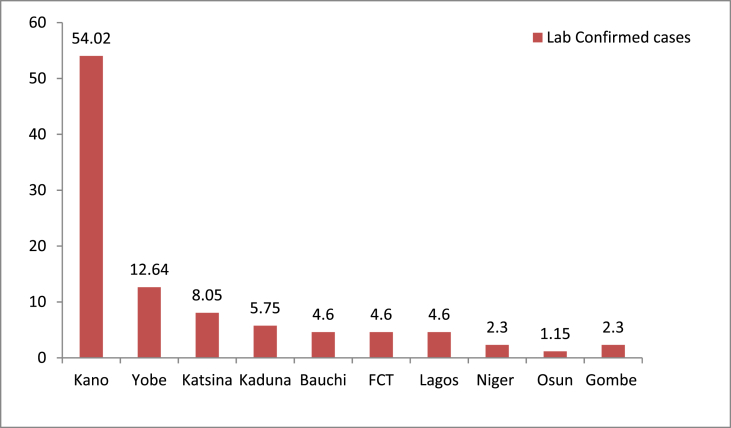
Laboratory confirmed cases of Diphtheria disease in some States in Nigeria.

As of 14 September 2023, the Nigeria Centre for Disease Control and Prevention (NCDC) had recorded 6,048 of the 10,077 suspected cases since the beginning of the year [8]. Kano State in north-west Nigeria is the most affected state in Nigeria, accounting for 85% of confirmed cases (Figure 2). Given the weak quarantine and porosity of the Nigerian borders, suspected cases have also been reported in Niger Republic. This may be due to the gradual spread of the disease to other states such as Bauchi, Borno and Yobe in the North-East [8]. Vaccination data have shown that more than 60% of suspected cases are unvaccinated, and as reported by Balakrishnan [9], the overriding factor in the diphtheria outbreak in Nigeria is the result of a historical gap in vaccine coverage. As of October 1, 2023, of the 8574 persons confirmed with diphtheria, 5476 (63.9%) were confirmed to be either unvaccinated or partially vaccinated, 2116 (24.6%) were fully vaccinated against diphtheria, and 769 (8.9%) had unknown vaccination status [9].

**Figure 2:**
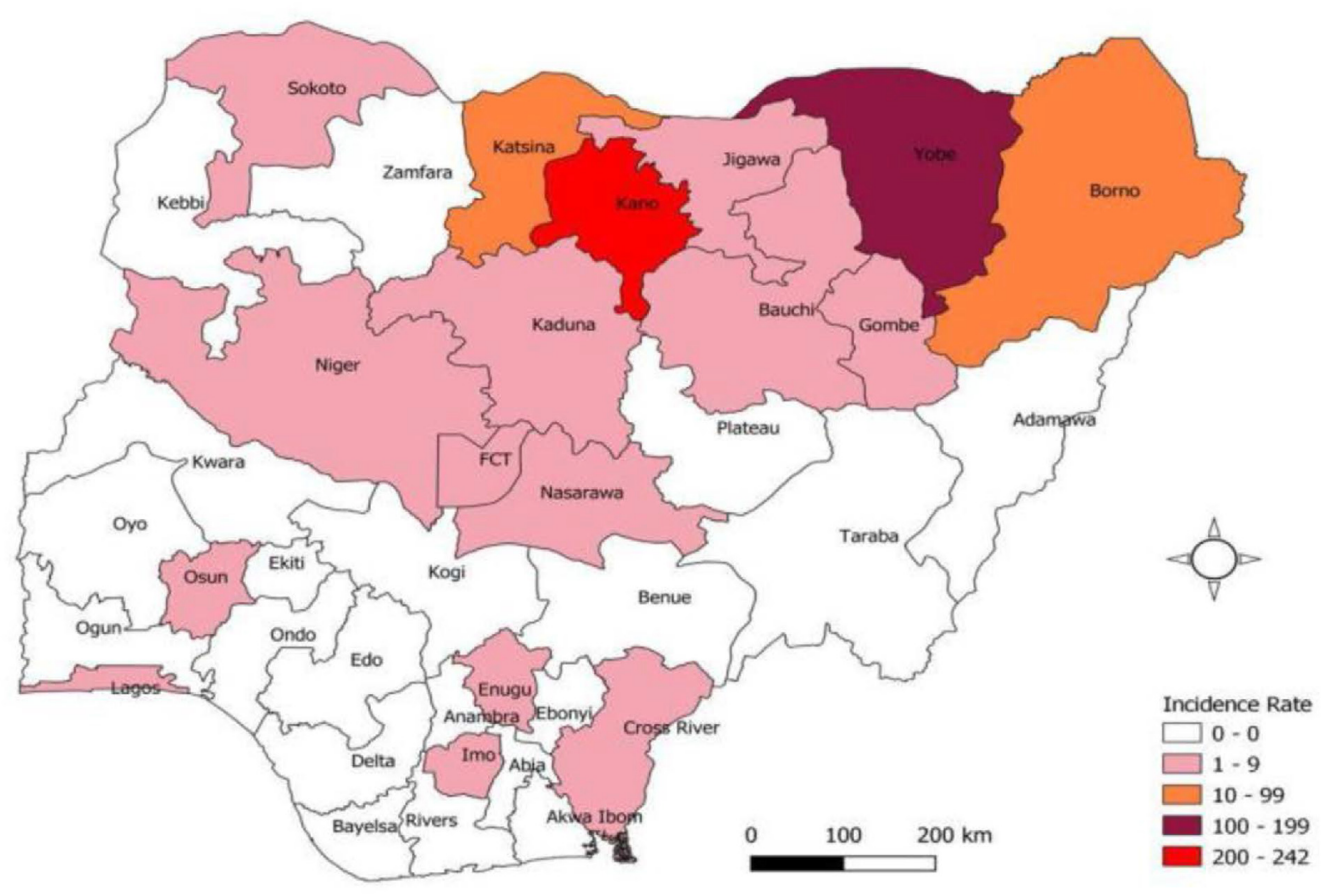
Diphtheria disease prevalence in some States in Nigeria as at 12 September 2023 (UNICEF: Situation Update, 2023).

On 14 September, 2023, the Minister of Health activated the Emergency Task Force to coordinate the response to the diphtheria outbreak. So far, 3 vaccination campaigns have been carried out in Kano State alone with another outbreak response scheduled to start on 25 September, 2023 and the second round vaccinations scheduled for 12 October, 2023 [8,10].

At present, the outbreak in Kano and Yobe states currently requires a total of 2.69 million doses of tetanus-diphtheria (Td) vaccine and 1.67 million doses of pentavalent (PENTA) vaccine [9]. There is currently a shortfall of 0.78 million doses of pentavalent vaccine [8]. UNICEF's situation report shows that about 2.4 million children have been vaccinated in four states; nearly 830,000 children in routine immunization (PENTA) and 1.6 million in outbreak response (Td) [8]. Currently, 12 million doses of PENTA vaccine have been secured by the Federal Government of Nigeria, and an additional 7 million doses of Td vaccine are in the pipeline. The strategy is to achieve 95% coverage with 3 primary doses of pentavalent (PENTA) vaccine in each LGA to control diphtheria [9]. The constraint of accessible and robust laboratory facilities across the country for reliable identification of toxigenic *Corynebacterium diphtheriae* and early detection of diphtheria outbreaks is a major challenge [8,10].

In conclusion, in order to stem the tide and contain the diphtheria outbreak the NCDC is working jointly with the United Kingdom Health Security Agency (UKHSA) to train health-care workers in diphtheria culture and set up infrastructural facilities to support existing ones. Presently, NCDC set up 14 laboratories in Nigeria to support diphtheria testing with the aim to optimize five more, with laboratory equipment being installed in Kaduna, Katsina, and Bauchi State.

## Conflicts of interest statement

No conflicts of interest related to this study and letter.

## Ethics approval

None.

## Financial support

None.
